# Technology-Enhanced Learning and Well-being: a Contribution to the Validation of a Measure to Assess University Students’ Technostress in the Italian Context

**DOI:** 10.1007/s11469-022-00940-9

**Published:** 2022-11-02

**Authors:** Giovanni Schettino, Leda Marino, Vincenza Capone

**Affiliations:** Department of Humanities, University of Naples, Federico II, Via Porta di Massa, 1, 80138 Naples, Italy

**Keywords:** Technostress, Students, Well-being, Technology-enhanced learning, Scale validation

## Abstract

COVID-19 has forced many universities to adopt widely technology-enhanced learning (TEL), highlighting the role of technostress as a risk factor for detrimental outcomes that may be prevented through the assessment with reliable tools. Thus, the present study aimed to test the psychometric characteristics of the Italian validation of the technostress scale by Wang, Tan, and Li. A self-report online questionnaire was completed by 915 participants (aged 18–33 years) attending an online university course during the health emergency. A subsample of 301 subjects (*M*_Age_ = 20.91, *SD* = 1.93) filled out the same questionnaire after a 3-month time interval to evaluate the test–retest reliability. Confirmatory factor analysis verified the one-factor structure of the scale, which was confirmed across academic courses considered (first-year and senior students). Moreover, the findings showed significant associations with the Italian Technostress Creators Scale and the Italian Mental Health Continuum–Short Form, as well as a satisfactory test–retest coefficient value supporting its validity and reliability. In light of the above, the study provides a useful instrument to evaluate technostress related to TEL and indications to implement preventive interventions for this type of stress by improving students’ experience with learning technologies.

Technostress was firstly described by Brod (1984) as an adaptive disease caused by the inability to deal with new computer technologies effectively and healthily. More specifically, technostress can be defined as “any negative effect on human attitudes, thoughts, behaviors, or body physiology that is caused directly or indirectly by technology” (Weil & Rosen, 1997, p. 5). Information and communication technologies (ICTs) play a pivotal role in technostress development and its manifestations (La Torre et al., 2019), representing potential sources of apprehension and anxiety or *techno-anxiety* (Marcoulides, 1989), which can result in *techno-phobia* (Rosen & Maguire, 1990), an aversion to computers usage. Furthermore, the relationship with these technologies can manifest in the form of extensive and compulsive usage or *techno-addition* (Tarafdar et al., 2020).

Moreover, technostress has been regarded as a type of stress (Salanova Soria, 2003) associated with a negative psychological state due to the usage of ICTs. This state is affected by the perception of a mismatch between demands and resources related to ICTs, leading to unpleasant psychophysiological activation and negative attitudes toward them.

From the first description of technostress in the 1980s, literature on this disease has increased by including the investigation of its association with the usage of different technologies such as mobile computing devices (Hung et al., 2015), collaborative tools (Jena, 2015), corporate management systems (Ragu-Nathan et al., 2008), and recently, social media (Brooks & Califf, 2017) by expanding the literature concerning the impact of these platforms on individuals’ well-being (Califano et al., 2022; Caso et al., 2020; Schettino et al., 2022a). In this regard, a large number of studies (Joo et al., 2016; Ragu-Nathan et al., 2008; Tarafdar et al., 2010) on technostress, across different fields, have documented several negative consequences both for individuals and their organizations (González-López et al., 2021). In particular, technostress has been identified as a strong predictor of anxiety, diminished concentration, irritability, memory loss, sleep disturbances, relationship issues with family, and malaise (Arnetz & Wiholm, 1997; Capone et al., 2021; Loh et al., 2021; Porter & Kakabadse, 2006; Salo et al., 2019; Schettino et al., 2022b; Thomée et al., 2012). At the organizational level, literature has widely recognized the role played by technostress as a risk factor for dissatisfaction with job, organizational commitment, decreased productivity, job burnout, absenteeism, and even intentions to quit the job (La Torre et al., 2020; Ragu-Nathan et al., 2008; Reinke et al., 2016; Tarafdar et al., 2007). In the educational environment, such *modern disease* (Brod, 1984) has been identified as a factor leading to concerns such as burnout, decreased learning engagement, and reduced performance (Jena, 2015; Wang et al., 2020). Among students, females and lower-grade individuals are more susceptible to experiencing burnout associated with greater technostress levels (Upadhyaya & Vrinda, 2021). Furthermore, such pressures among students are not only associated with the simple use of technology, but specifically, with a change of requirements in many aspects of learning (i.e., design and delivery of content, learning process features, and its assessment) resulting from the mentioned usage (Jung et al., 2012).

Universities are among the major educational organizations where ICTs related to education — or technology-enhanced learning (TEL) — are used widely to increase productivity and knowledge acquisition (Ragu-Nathan et al., 2008). In particular, technology helps enhance various processes within higher education institutions as well as the teaching and learning process (Bianchi & Caso, 2021).

The health emergency has significantly boosted technology adoption in most academic contexts. TEL has passed from being complementary to a mandatory educational methodology (Gaebel et al., 2021; Longmuir et al., 2021). Indeed, there has been a sudden adoption of distance learning by most universities worldwide, even in countries with low implementation rates of TEL in pre-pandemic times, such as Italy (European Commission, 2020; Eurostat, 2020). This country has been the second-worst affected country by Coronavirus after China during the initial spread of the virus, meaning that Italy was among the first countries in Europe to experience remote learning for all schools and universities in March 2020 (Sebastiani & Palù, 2020).

This switch from face-to-face to distance learning has allowed schools and universities to continue their educational programs. Nevertheless, at the same time, such a teaching mode has triggered the technostress process (Estrada-Muñoz et al., 2021; Galvin et al., 2022).

Indeed, undergraduates have experienced increased technostress which, in turn, has been identified as a risk factor for exhaustion (Alvarez-Risco et al., 2021), anxiety and depressive symptoms (Galvin et al., 2022), poorer academic performances (Upadhyaya & Vrinda, 2021), negative experiences regarding family life (González-López et al., 2021), and decreased mental well-being (Schettino et al., 2022b). This sort of chain reaction may be explained by taking into account the more demands — in terms of time, knowledge, skills, and psychological pressures — of TEL than the traditional learning mode (Commodari & La Rosa, 2021). In this regard, Penado Abilleira et al. (2021) recognized the person-environment (P-E) fit theory as an explanatory model of technostress among academic students. The scholars, using a sample of students from Spanish universities, have adapted a 20-item technostress scale from Chinese teachers to Spanish students following the P-E fit theory assumptions in order to provide a measure able to assess the prevalence of the disease in the considered population.

With the same aim, Wang and colleagues (2020) have developed and validated a more agile technostress measure consisting of 8 items specifically designed to assess university students’ technostress in TEL. They tested the psychometric properties of their tool with a sample of Chinese students from two public universities in China, demonstrating its validity as well as the fundamental contribution of P-E fit theory in understanding the development and outcomes of stress in various contexts.

The core of the P-E fit theory was identified by Edwards (1996, p. 292) in “the premise that attitudes, behaviors and other individual-level outcomes result not from the person or environment separately, but rather from the relationship between the two.”

Consequently, a good fit can promote well-being and increase job satisfaction and performance (Edwards & Rothbard, 1999; Gander et al., 2020; Gilbreath, 2004; Redelinghuys & Botha, 2016).

If the relationships between individuals and their environments are unbalanced, tensions could be generated, resulting in poor fits (Ayyagari et al., 2011; Edwards & Rothbard, 1999). Hence, stress occurs when there is no adjustment between the person and the environment or, in other words, between the resources of the person and the demands of the environment (Edwards et al., 1998). On the one hand, demands related to technostress can be defined as quantitative and qualitative requirements of technology-enhanced learning for individuals’ skills. On the other hand, supplies are different resources and opportunities that TEL can offer to meet the needs of individuals.

Consistent with the P-E fit theory, in Wang et al.’s (2020) scale, the *person* factor refers to university students, and the *environment* factor to technology-enhanced learning, its requirements, courses, and tasks. In the development process of Wang et al.’s (2020) instrument, the first-order 1-factor technostress scale with eight items was found to be psychometrically robust, demonstrating high internal consistency, validity, unidimensionality, and measurement invariance across participants of different demographics in China. Rapid assessments are regarded as efficient methods for collecting information quickly and when it is impossible to implement classical research methodologies as suggested in a pandemic. Therefore, in light of the studies mentioned above, the aim of this research was two-fold:

- Validating the technostress questionnaire by Wang et al. (2020) among Italian university students and corroborating its psychometric properties.
- Determining whether the theoretical perspective of the P-E fit theory was suitable to explain the prevalence of technostress and its association with well-being among Italian university students.

## Aims and Hypotheses

The current research aimed to evaluate the validity of the Italian Technostress Scale for University Students in TEL (IT-TSUS) by investigating the psychometric properties (factor structure, reliability, convergent validity, and discriminant validity) and its invariance across the courses of study (first year, students who had been enrolled at university for less than a year; third year, students who had almost finished the first round of the course). A further aim was to expand the evidence about the tool by examining the relationships between IT-TSUS and some criterion variables such as emotional, social, and psychological well-being.

Consequently, we formulated the following hypotheses.

- **Hypothesis 1a (H1a)**. *We expected to confirm the one-factor structure of IT-TSUS as found in Wang and colleagues’ tool*.
- **Hypothesis 1b (H1b)**. *We hypothesized that the IT-TSUS had high internal reliability, similar to earlier findings in the Chinese sample*.
- **Hypothesis 2a (H2a)**. *We hypothesized that our study confirmed the convergent validity of the IT-TSUS correlating positively with corresponding measures. Thus, we expected the IT-TSUS to correlate positively with technostress at work*.
- **Hypothesis 2b (H2b)**. *Considering divergent validity, we expected that IT-TSUS correlated negatively with mental well-being*.
- **Hypothesis 3 (H3a)**. *We expected an adequate test–retest reliability of the IT-TSUS*.
- **Hypothesis 4 (H4)**. *Referring to group invariance, we hypothesized that the functioning of the IT-TSUS items did not differ across academic courses (first-year students of psychology course and senior group, in the third year of psychology course)*.

## Material and Methods

### Translation Process

The IT-TSUS was back-translated to ensure translation equivalency. Psychometric testing of the *P-E fit scale of technostress for university students in technology-enhanced learning* with an Italian sample was then conducted. Two bilingual Ph.D. researchers participated in the translation process. One of the researchers, who translated the original tool into Italian, had gotten a Ph.D. in Health Psychology in Italy. The second translator had been educated in the United States and had a Ph.D. in Psychology. He translated the Italian TSUS back to English without discussion with the first researcher. Subsequently, adjustments were performed in order to ensure understandability, psychological equivalence, and the accuracy of the translation from English into Italian. As a result, the original and back-translated English versions did not differ significantly, as posited by the translators. Lastly, on the basis of feedback from six university students, the wording and clarity of the description of the scale items were improved.

### Procedure and Statistical Analyses

We used a cross-sectional design to validate the IT-TSUS. Participants were recruited among psychology students attending an online course at the University of Naples Federico II in March 2021. They were invited to complete an online self-report questionnaire about their experience with technology-enhanced learning. Research participation was voluntary, and respondents received no reward. Moreover, it was subjected to the privacy information and consent to process personal data following the applicable law. No outliers were identified, and no missing values were found since all the answers were mandatory.

Bartlett’s test of sphericity and the Kaiser–Meyer–Olkin (KMO) measure of sampling adequacy were used to test whether the dataset was suitable for factor analysis. In order to analyze the reliability of the scale, we computed the internal consistency using Cronbach’s alpha. Furthermore, for the analysis of the scale internal consistency, the corrected correlations between the score of the items and the IT-TSUS, as well as test-rest reliability, were analyzed.

As recommended by researchers in the field (Byrne et al., 1989), the following steps were taken to test factor invariance. Preliminary confirmatory factor analysis (CFA) was performed using the maximum likelihood estimation method to analyze the underlying structure of items. To evaluate the solution, we took into account the goodness of fit indexes. Thus, the chi-square (*χ*^2^) was computed to examine the difference between observed and expected covariance matrices by testing the null hypothesis of ideal model fit where the residual covariance equals zero. We also considered adequate comparative fit index (CFI) and the Tucker–Lewis’s index (TLI) values above 0.90 (Bentler, 2006; Byrne et al., 1989), root-mean-square error of approximation (RMSEA) values below or equal to 0.06, and root-mean-square residual (RMSR) values equal to or below 0.09 (Hu & Bentler, 1999). Furthermore, the Pearson product-moment correlations were computed to examine the relations between the measures. Statistical significance was set at *p*-value < 0.05. This CFA was performed separately for the first-year and senior students groups to assess whether the model fitted the data well in each group. Then, multi-group CFA was conducted in order to test configural (Thurstone, 1947) and metric invariance (Millsap & Olivera-Aguilar, 2015).

### Participants

The sample consisted of 915 Italian students (83.9% women) aged 18–33 years (*M* = 20.81; *SD* = 1.98). Of the respondents, 40% (*N* = 366) attended the first year of a psychology degree, whereas 60% (*N* = 549) attended the last year of the course. The most were unemployed (83.6%; *N* = 765), 485 (53%) were single, and 430 (47%) were engaged. About participants’ experience with TEL, 83.7% (*N* = 766) had a place in their houses only for it, and most (41.2%; *N* = 377) reported there was one other person besides them who used TEL or worked in smart mode in their houses, 189 (20.7%) answered two people, 167 (18.2%) declared more than two people using online learning or working, while 182 (19.9%) respondents indicated they were the only ones in their families. Lastly, in order to assess the test–retest validity, a subsample consisting of 301 students (*M*_Age_ = 20.91, *SD* = 1.93; 84% female) completed the questionnaire again after three months. Ethical approval for the current study had been obtained from the Department of Humanities Ethical Committee of Psychological Research prior to the commencement of this project. All participants provided informed consent.

### Measures

The Italian version of TSUS (IT-TSUS): The tool consists of 8 closed questions (e.g., “I feel stressed to adapt to technology-enhanced learning”) assessing university students’ technostress. Response alternatives are provided on a 5-point Likert-type scale from 0 (“strongly disagree”) to 4 (“strongly agree”). In this study, *α* = 0.95.

Measure for convergent validity. The technostress creators scale (TCS; Molino et al., 2020; Ragu-Nathan et al., 2008) is an 11-item Likert scale that measures workers’ technostress. The instrument consists of three factors: techno-overload (4 items, e.g., “I am forced by technology to work much faster”; *α* = 0.82), techno-invasion (3 items, e.g., “I spend less time with my family due to technology”; *α* = 0.71), and techno-complexity (4 items, e.g., “I do not know enough about technology to handle my job satisfactorily”; *α* = 0.89). Answers are ranged on a 5-point Likert scale from “strongly disagree” (1) to “strongly agree” (5). Cronbach’s alpha for The Technostress Creators scale was 0.85.

Measures for divergent validity. The Italian Mental Health Continuum Short Form (MHC-SF; Petrillo et al., 2015) consists of 14 statements measuring psychosocial well-being as described in Keyes’ model (Keyes, 2002). It is a scale assessing emotional (e.g., “During the past month, how often did you feel satisfied with life”; *α* = 0.86), social (e.g., “During the past month, how often did you feel that you belonged to community”; *α* = 0.79), and psychological well-being (e.g., “During the past month, how often did you feel that you have experiences that challenge you to grow and become a better person”; *α* = 0.85). The MHC–SF asks individuals how much of the time they functioned in a specific manner, from 0 (“none of the time”) to 5 (“all of the time”). MHC-SF reported a Cronbach’s alpha of 0.91.

Demographic data. Participants were asked to specify their age, gender, nationality, marital status, and employment status.

## Results

### Descriptive Statistics and Confirmatory Factor Analysis

Descriptive analyses, Pearson’s correlation, and computation of Cronbach’s alpha coefficients were performed using SPSS 27 statistical software (IBM Corp., Armonk, NY, USA). In addition, confirmatory factor analysis (CFA) was carried out through the support of Mplus 8.6 (Muthén & Muthén, Los Angeles, CA, USA). Descriptive statistics for the IT-TSUS and the other psychological variables assessed are presented in Tables 1 and 2.

**Table 1:** Descriptive statistics of items of IT-TSUS (*N* = 915). In brackets, the original English items are reported

	**Item**	**Mean**	**SD**	**Skewness**	**Kurtosis**
ITEM 1	*Adattarmi alla didattica a distanza mi fa sentire sotto pressione* [I feel stressed to adapt to technology-enhanced learning]	2.45	1.26	.05	− .67
ITEM 2	*Ho difficoltà a seguire in maniera efficace la didattica a distanza perché ho poco tempo e risorse* [I find it difficult to effectively use technology-enhanced learning due to my limited investment of time and effort]	2.17	1.24	.29	− .55
ITEM 3	*Mi sento stressato/a dalle elevate richieste della didattica a distanza alle quali non riesco a far fronte con le mie attuali capacità* [I feel stressed to cope with the high demands of technology-enhanced learning with my current capability]	2.32	1.24	.22	− .63
ITEM 4	*Mi vedo costretto/a cambiare le mie abitudini e scelte di apprendimento per adeguarmi alle richieste della didattica a distanza* [I am pressured to change my current learning habit and preference to meet the requirements of technology-enhanced learning]	2.74	1.22	− .30	− .50
ITEM 5	*La didattica a distanza invade diffusamente ogni aspetto della mia formazione, creandomi disagio* [I am not comfortable with the pervasive invasion of technology-enhanced learning in all aspects of my study]	2.15	1.22	.32	− .49
ITEM 6	*Sono infastidito/a dalle varie forme della didattica a distanza* [I am irritated by the vast variety of technology-enhanced learning]	2.17	1.22	.22	− .57
ITEM 7	*Mi stressano le varie forme di didattica a distanza, perché rendono più difficile il mio studio* [I feel stressed as the various forms of technology-enhanced learning complicate my study]	2.32	1.26	.16	− .68
ITEM 8	*Mi sento stressato/a perché la diffusione della didattica a distanza ha portato disordine nelle mie abitudini di studio* [I feel stressed as the heavy reliance on technology-enhanced learning in my school disrupts my normal study pattern]	2.52	1.30	− .03	− .74

**Table 2:** Bivariate correlations with validation measures (*N* = 915)

	**M (SD)**	**IT-TSUS**	**1**	**2**	**3**	**4**	**5**	**6**	**7**
**IT-TSUS**	2,355 (1.058)								
1. MHC-SF	2,379 (.884)	− .184^**^							
2. MHC-SF—Emotional well-being	2.646 (1.099)	− .216^**^	.843^**^						
3. MHC-SF—Social well-being	1.585 (.956)	− .095^**^	.834^**^	.591^**^					
4. MHC-SF—Psychological well-being	2.908 (1.028)	− .179^**^	.909^**^	.698^**^	.581^**^				
5. TCS	2.129 (.872)	.638^**^	− .170^**^	− .175^**^	− .091^**^	− .177^**^			
6. TCS—Techno-overload	3.051 (.867)	.575^**^	− .141^**^	− .138^**^	− .087^**^	− .141^**^	.804^**^		
7. TCS—Techno-invasion	3.207 (.943)	.533^**^	− .102^**^	− .118^**^	− .075^*^	− .083^*^	.781^**^	.548^**^	
8. TCS—Techno-complexity	2.129 (.872)	.371^**^	− .143^**^	− .144^**^	− .047	− .174^**^	.722^**^	.293^**^	.327^**^

Note. *p < .05; **p < *.01*

**Fig. 1:**
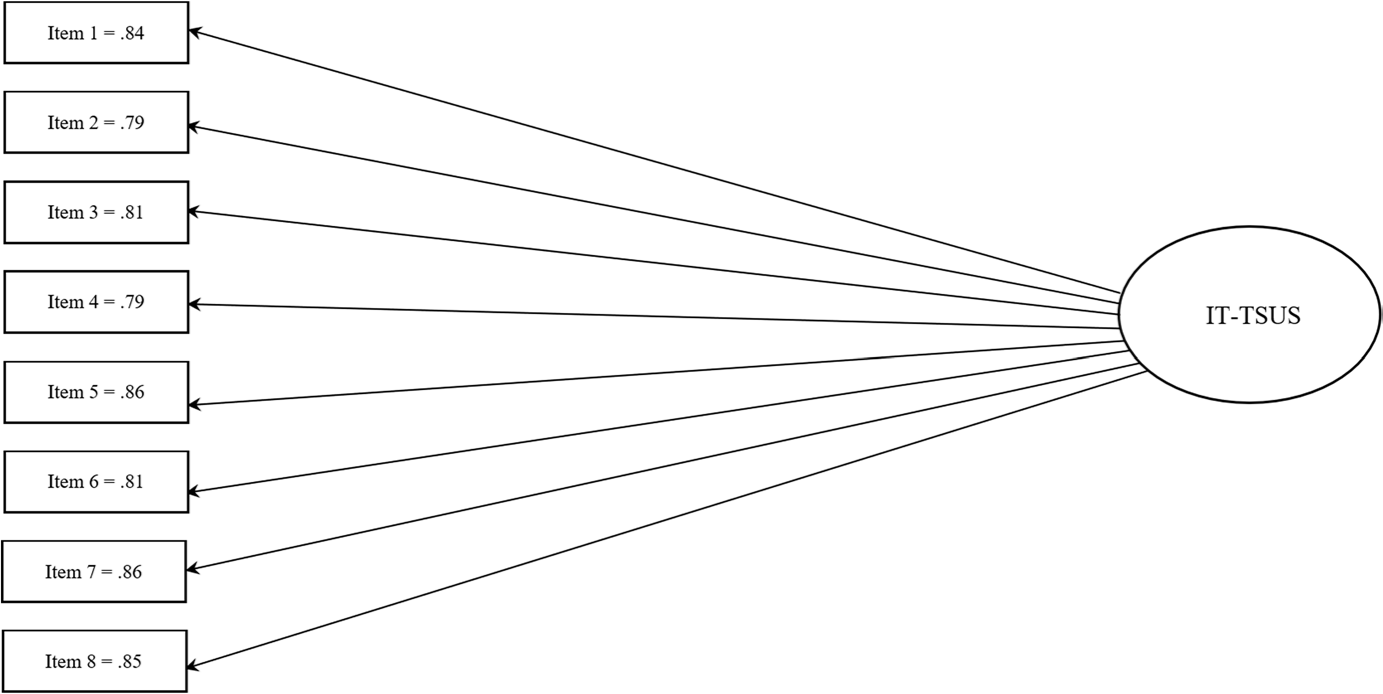
Structure of IT-TSUS. Standardized coefficients

Analysis of the IT-TSUS individual items indicated that item scores were not skewed, with none of them showing extreme means and close to zero variances. The average score obtained in the IT-TSUS was 2.36, *SD* = 1.06 (Table 3). In order to test the internal consistency of the IT-TSUS, each item score was correlated with the total score of the scale. Statistically significant part-whole correlations were reported. The coefficient range was between 0.79 and 0.86 (Fig. 1). Since they were greater than 0.30, the internal consistency of the IT-TSUS can be considered good (Nunnally, 1994). Furthermore, CFA was performed to ascertain the factor structure of the scale.

Bartlett’s sphericity test was equal to *χ*^2^ (*df* = 28, *N* = 915) = 6077.632 (*p* < 0.001), and the KMO index was 0.94, indicating an adequate correlation matrix. The computation of skewness and kurtosis indices (Table 1) showed the normality of the distribution. Kurtosis ranged between − 0.74 (item 8) and − 0.49 (item 5); skewness ranged between − 0.30 (item 4) and 0.29 (item 2). Similar to the findings reported by Wang, Tang, and Li (2020), who identified a one-factor structure, one factor with eigenvalues greater than one emerged in our study.

The one-factor model fitted the data best. The fit statistics for the model were as follows: *χ*^2^ = 59.699 (33, *N* = 915), exact *p* = 0.02; CFI = 0.96; TLI = 0.094; RMSEA = 0.081; 90% confidence interval for RMSEA = 0.049, 0.08; SRMR was 0.025. Standardized factor loadings of items ranged from 0.79 to 0.86 (see the Appendix). The single factor explained 72.23% of the variance. All residual correlations were lower than |.1|. Finally, the scale reported good internal reliability. The Cronbach’s alpha of the Italian TSUS was 0.94.

### Convergent and Divergent Validity

A significant positive correlation between IT-TSUS scores and those on the technostress creators scale (*r* = 0.64, *p* < 0.01) confirmed the convergent validity of the scale. More specifically, a high and positive correlation was reported with the *overload* dimension (*r* = 0.58*, p* < 0.01) and *invasion* dimension (*r* = 0.53*, p* < 0.01) of the TCS. In addition, the IT-TSUS correlated negatively with the MHC-SF (*r* =  − 0.18, *p* < 0.01) and its dimensions.

### Crossover Path Analysis (Test–Retest)

To examine the stability of the scale, we performed a crossover path analysis of the IT-TSUS later in time, considering a reduced sample (*N* = 301). We correlated the time points March 2021 (t0) and June 2021 (t1). Findings demonstrated that the initial results predicted those at the follow-up (*r* = 0.71; *p* = 0.01). Besides, the intraclass correlation coefficient (ICC) for IT-TSUS was 0.64 (*p* < 0.0), suggesting good reliability of the scale (Shrout & Fleiss, 1979).

### Testing for Factor Invariance

Some procedures were accomplished to test the factorial invariance of the 8-item version of the IT-TSUS. Firstly, we performed a preliminary confirmatory factor analysis, in which the single factor was posited separately for first-year students and senior groups. The model taken into account fitted the data well in each group: first-year students: *χ*^2^ = 91.114 (39), *p* < 0.001; CFI = 0.95; TLI = 0.93; RMSEA = 0.07 (0.062 0.088); SRMR = 0.025; senior: *χ*^2^ = 77.516 (39), *p* < 0.001; CFI = 0.95; TLI = 0.93; RMSEA = 0.07 (0.056 0.075); SRMR = 0.034. Multigroup CFA was subsequently conducted to examine configural invariance (Thurstone, 1947). The model fitted the data well: *χ*^2^ = 210.470 (86), 0.000; CFI = 0.943; TLI = 0.932; RMSEA = 0.07 (0.72 0.85); SRMR = 0.048. Standardized factor loadings of items were all significant. Coefficients ranged from 0.75 to 0.90 in the first-year group and from 0.71 to 0.91 in the senior group. Finally, we investigated metric invariance and found this model to be tenable: *χ*^2^ = 210.589 (130), *p* < 0.001; CFI = 0.932; TLI = 0.930, RMSEA = 0.07 (0.075 0.089); SRMR = 0.040. Thus, we compared Model 0 with a model that was the same, except all latent means were fixed to be equal across groups. A non-significant drop in model fit was observed, indicating that latent mean differences did not exist between gender groups (Δ CFI =  − 0.001, *p* = 0.40).

## Discussion

The COVID-19 pandemic has disrupted any aspect of our lives. In an effort to control the spread of the virus, worldwide governments have imposed restrictive measures. Luckily, the negative consequences of this pandemic have been partially reduced by technology which has allowed individuals to shift from offline to online several human activities such as meeting, entertaining, working, and learning. In the high school sector, universities have continued their activities through TEL. This challenge has been difficult to deal with, particularly for those countries unprepared for online learning, such as Italy (European Commission, 2020; Preply, 2021). Due to the health emergency, Italian students have been among the first to face a sudden shift from face-to-face learning to distance learning (Sebastiani & Palù, 2020). In such a situation, the risk of suffering from technostress is high. Therefore, it is evident that context-sensitive tools should be used for adequate identification of TEL psychological indicators. Following this line of reasoning, the present study aimed to validate the Italian version of the technostress scale for university students in TEL (Wang et al., 2020) in order to develop a brief and valid assessment measure of technostress.

The findings were very satisfactory, supporting all hypotheses made. Indeed, the IT-TSUS showed psychometric properties similar to the original Chinese version. The CFA highlighted the one-factor dimension (H1a) similarly to the measure of Wang and colleagues (2020). Moreover, the scale reported good reliability (H1b), convergent validity (H2a), discriminant validity (H2b), and stability over time (H3). Furthermore, the invariance calculation suggests that the scale could be used both among university students at the beginning of their academic careers and students about to graduate (H4). Starting from above, the IT-TSUS can be regarded as a suitable and agile instrument for evaluating students’ stress related to TEL. In addition, the results highlighted above-average levels of technostress among the university students enrolled in the research. Given their age (*M*_age_ = 20.81), they can be regarded as Digital Natives (Prensky, 2001; Rothman, 2016) because they have been growing up through immersion in digital technology (Thompson, 2013). As a result, they are habituated to quick and autonomous access to information, multitasking, nonlinear learning, and dynamic graphics (Brooks & Davis, 2018, Thompson, 2013). However, these digital skills have not protected them from technostress related to distance learning. Specifically, our findings suggest that technostress may have been a risk factor for these Italian Digital Natives’ well-being during the COVID-19 pandemic. The participants who reported higher stress about remote learning were those who experienced decreased mental well-being. The P-E fit theory (Edwards, 1996) could offer a possible explanation for this result, suggesting that such students have felt unable to deal with changes related to the quick adoption of distance learning, perceiving a stress condition. Moreover, the theoretical framework indicates that determinants of this perception should be found in both students and their universities. Thus, it seems they did not have adequate individual resources for this challenge, and their university did not make a proper valuation of the student’s capability to cope effectively with TEL.

The findings of this study should be interpreted cautiously and considered in the context of some limitations which can be addressed in future research. More in detail, the study was conducted during a pandemic crisis, so its results could be influenced by factors associated with a period of special stress among students and academic organizations (Galvin et al., 2022; Procentese et al., 2020), unprepared to deal with the unprecedented situation. Besides, participants were recruited from the first and third years of a specific degree, making it difficult to generalize the results obtained to the more general academic population. Finally, since we adopted a self-report questionnaire, it is possible that data could be affected by the common method invariance issue. Therefore, future studies should take a longitudinal approach to test the scale reliably over time. In particular, the tool should be tested once the health emergency situation will have been overcome.

Despite the limitations mentioned above, the good psychometric properties of the scale suggest a wide use of the instrument for timely identification of students at higher risk of developing technostress and negative consequences related to this modern disease. Moreover, the study supports the P-E fit theory ability to investigate students’ technostress. As far as we know, our research is the first one to adopt such a theoretical framework in the Italian context to examine university students’ technostress caused by distance learning adoption. Based on the strong psychometric properties of the scale, we recommend extensive use of the IT-TSUS in research with students and health interventions. Furthermore, the scale should also be tested and validated in languages other than Italian for usage across cultures. Universities and public organizations around the globe should pay closer attention to the role of technostress at the individual level in more effectively managing policies and interventions. For this purpose, we believe the IT-SUS proved to be a tool able to provide useful information in designing online learning contexts which can improve students’ well-being. Specifically, such interventions should enhance students’ resources, for example, their technological skills, to make them more effective in coping with TEL-related demands and preserving their mental health.

## Appendix

Table 3

**Table 3:** Loading of the items of IT-TSUS, corrected correlations, and items reliability (*N* = 915)

	**Factor loading**	**Item-total corr. correlation**	***α***** if deleted**
ITEM 1	.838	.81	.937
ITEM 2	.786	.76	.940
ITEM 3	.811	.79	.938
ITEM 4	.793	.77	.940
ITEM 5	.863	.83	.935
ITEM 6	.807	.78	.939
ITEM 7	.857	.83	.936
ITEM 8	.853	.83	.936
